# A novel protocol to detect green fluorescent protein in unfixed, snap-frozen tissue

**DOI:** 10.1038/s41598-020-71493-x

**Published:** 2020-09-04

**Authors:** Valentina Scandella, Rosa Chiara Paolicelli, Marlen Knobloch

**Affiliations:** Department of Biomedical Sciences, University of Lausanne, Rue du Bugnon 7, 1005 Lausanne, Switzerland

**Keywords:** Biological techniques, Immunological techniques, Immunohistochemistry, Neuroscience

## Abstract

The green fluorescent protein (GFP) is a powerful reporter protein that allows labeling of specific proteins or entire cells. However, as GFP is a small soluble protein, it easily crosses membranes if cell integrity is disrupted, and GFP signal is lost or diffuse if the specimen is not fixed beforehand. While pre-fixation is often feasible for histological analyses, many molecular biology procedures and new imaging techniques, such as imaging mass spectrometry, require unfixed specimens. To be able to use GFP labeling in tissues prepared for such applications, we have tested various protocols to minimize the loss of GFP signal. Here we show that, in cryocut sections of snap-frozen brain tissue from two GFP reporter mouse lines, leaking of the GFP signal is prevented by omitting the commonly performed drying of the cryosections, and by direct post-fixation with 4% paraformaldehyde pre-warmed at 30–37 °C. Although the GFP staining does not reach the same quality as obtained with pre-fixed tissue, GFP localization within the cells that express it is preserved with this method. This protocol can thus be used to identify GFP positive cells on sections originating from unfixed, cryosectioned tissue.

## Introduction

Reporter proteins are helpful tools used in a wide range of applications in biology. One of the most frequently used is the green fluorescent protein (GFP), originally extracted from the jellyfish *Aequorea Victoria*^1,2^*.* GFP is a small protein of 238 amino acids (27 kDa) that can be easily engineered and detected. More than 40′000 published articles have used GFP in one way or another, since the first breakthrough in 1994^3^. Despite serving as a great tool to tag various proteins and organelles, certain limitations remain: (i) GFP fluorescence gets weakened or lost upon specific fixation conditions^4^. As there are good antibodies available, this problem can usually be overcome by staining against GFP to enhance the signal. (ii) GFP is a soluble protein that can cross the cell membrane when it loses its integrity. This can cause problems when GFP is not tethered to any other protein, as is the case for many transgenic reporter constructs, where GFP is expressed under the promoter of a gene of interest and remains freely in the cytosol once expressed^5^. Indeed, it has been previously shown that GFP protein is lost in the washing steps during immunostaining^6^. To overcome this issue, cells or tissues require fixation to crosslink GFP to other proteins or macromolecules prior such procedures^4,7^. When using 4% paraformaldehyde (PFA), one of the most common fixatives, GFP crosslinks and remains detectable where it was initially expressed.

While these solutions are applicable for many situations, therefore not posing a major problem, certain applications require unfixed cells or tissues for downstream processing. This applies especially to techniques aimed at the visualization of metabolic enzymatic activities (metabolic mapping^8^) or molecules directly on tissues sections. Imaging mass spectrometry (IMS) for instance, requires the preservation of all the macromolecules and metabolites without fixatives or any cryopreservative solution, which is usually achieved by snap freezing samples^9,10^. As these techniques are preserving the spatial relationship, histological information can be overlaid on the obtained data. Thus, performing such studies on GFP labelled tissues would be highly informative. However, as the tissue has to remain unfixed prior to sectioning, GFP detection and accurate localization becomes problematic, due to its leakiness out of the cells.

We thus aimed at optimizing a protocol for improving the GFP signal in unfixed, snap-frozen tissue sections. To do so, we have compared several post-sectioning fixation methods on brain tissue of the Nestin-GFP reporter mouse^11^, expressing GFP under the control of the Nestin promoter. In this mouse, GFP is expressed in neural stem/progenitor cells (NSPCs), for instance in the dentate gyrus (DG) of the hippocampus. We show that fixation methods crucially affect the quality and the localization of the GFP signal in unfixed, snap-frozen tissue. By using the Cx3cr1-GFP reporter mouse^12^, we also show that this optimized protocol works in different GFP mouse lines and can be used for other tissues, such as the lungs. Thus, we provide a protocol that can be used in adjacent sections to obtain histological GFP information in procedures requiring unfixed tissue sections.

## Results

**Figure 1:**
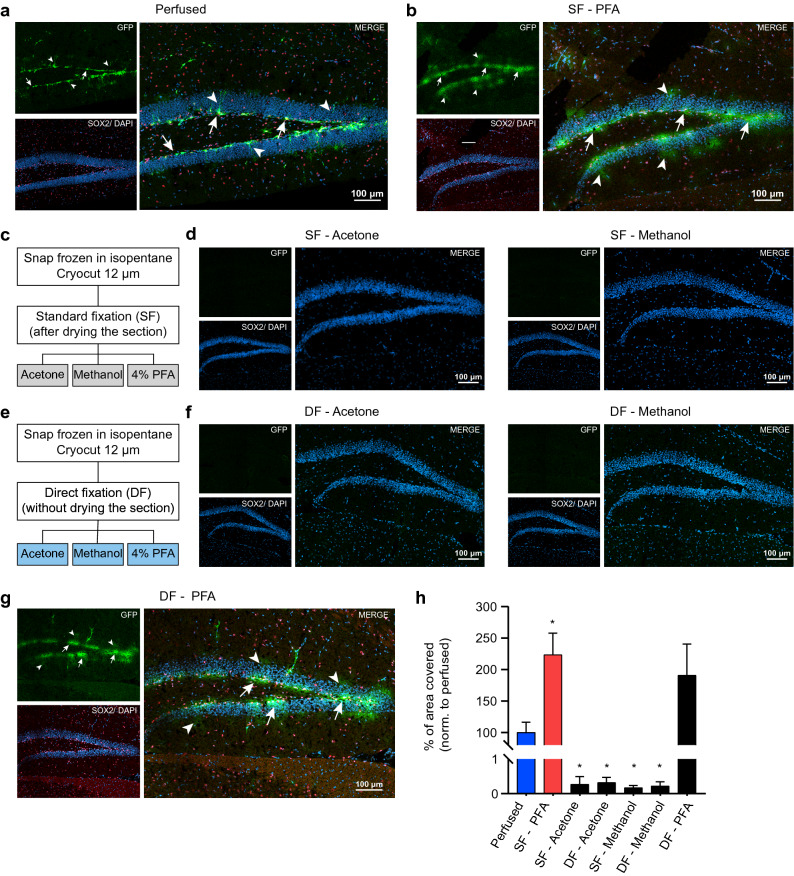
Comparison between a standard fixation and a direct fixation protocol on snap-frozen brain sections. **(a)** Co-staining of GFP (green, cytosolic) and SOX2 (red, nuclear) on a brain section from a perfused Nestin-GFP mouse show NSPCs with distinct cell bodies (arrows) in the subgranular zone of the DG and some radial processes (arrowheads). **(b)** Unfixed snap frozen brain sections that were post-fixed with 4% PFA using a standard protocol, show a diffuse GFP signal without clear cell bodies (arrows) and radial processes (arrowheads). However, SOX2 staining is localized in nuclei. **(c)** Schematic illustration of the different protocols used to improve the staining. In the standard fixation (SF) protocol, the cryosection is allowed to air-dry on the slide before continuing with any post-fixatives. Using this SF, cold acetone, cold methanol or 4% PFA were tested on unfixed snap frozen cryosections. **(d)** Co-staining of GFP and SOX2 on sections processed as described in c with either acetone (left) or methanol (right). With these two fixatives, both GFP and SOX2 signal are lost. **(e)** Schematic illustration of the different protocols with a direct fixation (DF) without letting the sections to air-dry. Post-fixatives are the same as used in c. **(f)** The DF with acetone (left) or methanol (right) does not improve the staining, and no signal was detectable. **(g)** Post-fixation using the DF protocol with 4% PFA allows the detection of GFP and SOX2 signal on unfixed snap frozen cryosections. GFP signal is localized to the subgranular zone of the DG (arrows) and improved compared to the SF protocol, however still diffuse. **(h)** Quantification of GFP signal per DG. The bar graph represents the percentage of GFP area normalized to the perfused condition (mean ± SEM, n = 3 sections per condition) that covers the image. For all images, a maximum projection of a confocal stack with the individual channels and a merge is shown. DAPI is used to mark nuclei. *p < 0.05 (one-way ANOVA followed by Holm-Sidak’s multiple comparison, compared to perfused).

The classical way of revealing GFP in Nestin-GFP mice is by using antibodies against GFP on brain tissue from transcardially-perfused and fixed animals. This method indeed enables to nicely visualize GFP-positive NSPCs (Fig. 1a). These cells, co-stained against the transcription factor sex determining region Y-box 2 (SOX2), are distinguishable in the subgranular zone (SGZ) of the DG, with the majority of GFP-positive cells having a radial process which spans the granular zone. However, immunostaining against GFP on cryosections from snap-frozen brain which have only been post-fixed (4% PFA at 25 °C), results in much less defined signal (Fig. 1b). In this case, although GFP is still located in the SGZ of the DG, it is quite diffused and individual cells are no longer distinguishable. In contrast, the SOX2 staining is located to nuclei and clearly distinct, suggesting that the post-fixation of snap-frozen, unfixed sections with 4% PFA is sufficient to crosslink nuclear proteins but not free-floating cytoplasmic GFP (Fig. 1b).

To test whether other fixatives than PFA would result in better GFP signal in such snap-frozen, unfixed cryosections, we used two chemical fixatives that are routinely applied in immunohistochemistry, namely pre-cooled acetone and pre-cooled methanol. When performing immunostaining against GFP and SOX2 on such post-fixed cryosections, almost no signal was detectable (Fig. 1c,d,h).

Cryosections are frequently allowed to air-dry at 25 °C after sectioning and mounting, to ensure a good attachment of the tissue to the glass slides before immunostaining. As this standard fixation (SF) procedure gave unsatisfactory GFP signal, we reasoned that during the drying step, unfixed GFP might leak out due to damaged cell membranes. We thus next tested whether the same three post-fixation methods would lead to better results when sections are directly immersed in fixative after mounting the frozen section on glass slides, omitting the drying step (Fig. 1e). While such direct fixation (DF) did not improve the GFP signal when using acetone and methanol (Fig. 1f,h), it did indeed improve the GFP signal compared to SF when 4%PFA was used as a post-fixative (Fig. 1g). The GFP signal was more confined, however still less sharp than on pre-fixed tissue. Quantification of the percentage of area covered by GFP among the different protocols confirmed that, among all the fixatives, PFA best preserved the signal when compared to brain sections from a PFA-perfused mouse. (Fig. 1h). Moreover, qualitative examination of SF-PFA and DF-PFA indicates that omitting the drying step slightly reduced the spread of the signal (Fig. 1b,g). Overall, these data are consistent with previous reports showing that GFP leaks out of the cells when the tissue is unfixed and frozen^4,6,7^.

**Figure 2:**
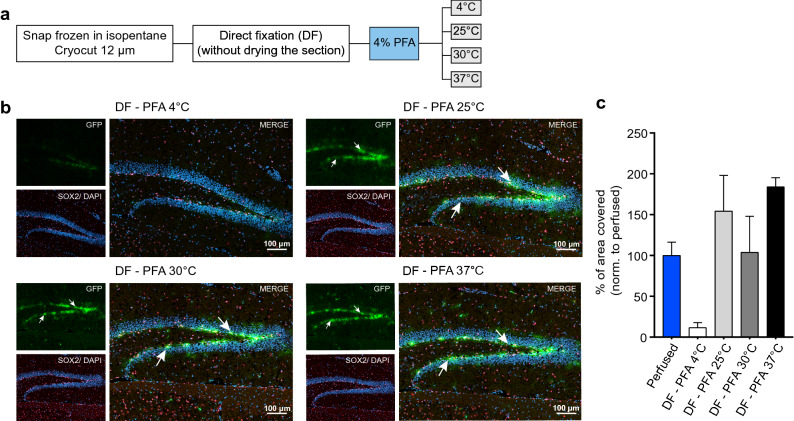
Temperature of PFA influences the post-fixation of GFP in snap-frozen brain sections. (**a**) Schematic illustration of the DF protocol using PFA at different temperatures for post-fixation. (**b**) Co-staining of GFP (green) and SOX2 (red) on snap frozen sections post-fixed with 4% PFA at 4 °C, 25 °C, 30 °C or 37 °C. Brain sections post-fixed with PFA at 4 °C do not show any GFP signal, whereas SOX2 staining is maintained in the nuclei. GFP fluorescence is detectable in sections post-fixed with PFA at 25 °C, although as a diffuse signal. By increasing the temperature of PFA to 30 °C or 37 °C, the GFP signal is stronger and more confined compared to brain sections post-fixed with PFA at 25 °C (arrows). Shown is a maximum projection of a confocal stack with the individual channels and a merge. (**c**) Quantification of the GFP signal per DG. The bar graph represents the percentage of GFP signal that covers the DG normalized to the perfused condition (mean ± SEM, n = 3 sections per condition). The blue bar is taken from the graph displayed in Fig. 1h to facilitate comparisons. One-way ANOVA (significant), followed by Holm-Sidak’s multiple comparisons: non-significant. However unpaired t-test between the following groups showed a significance: perfused vs DF- PFA 4 °C: p = 0.007, perfused vs DF-PFA 37 °C p = 0.01.

The improvement of the GFP signal with the DF-PFA protocol indicates that the leaking of GFP most likely occurs during the drying step of the unfixed cryosections. This step involves a major temperature shift in the section, from – 20 to 25 °C. Leaking of GFP is most likely increased at higher temperatures, thus crosslinking should occur as quick as possible while the section is thawing. As the rate of penetration of PFA is temperature-dependent^13^, we next tested whether different temperatures of the 4% PFA solution could improve the DF protocol. While higher temperatures of PFA should result in quicker fixation, they also speed up the thawing of the section, whereas cold PFA should decrease the temperature difference between section and solution, however resulting in slower fixation. As it is unclear which aspect is the most important for the GFP leakiness, we tested temperatures of PFA ranging from 4 to 37 °C (Fig. 2a).

**Figure 3:**
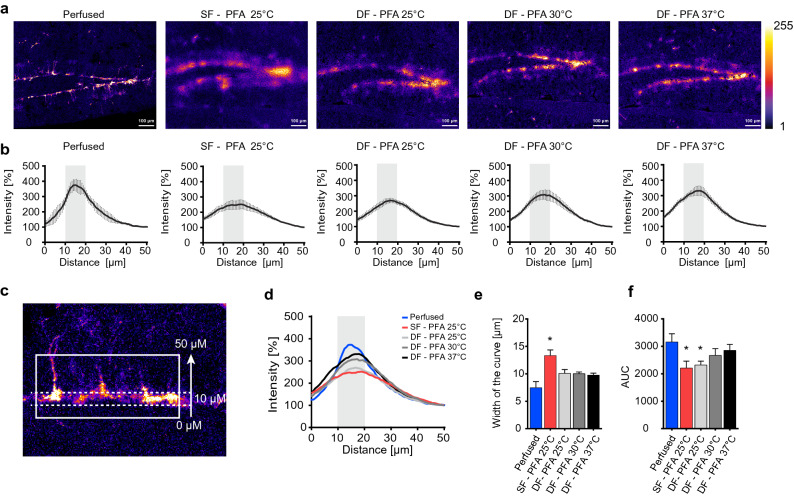
An increase in PFA temperature allows a more confined GFP signal in snap frozen brain sections. (**a**) Confocal stack images are converted into a false color intensity mapping. Sections fixed with the DF protocol show a more defined GFP signal compared to sections processed with the SF protocol. (**b**) GFP intensity plots across the granular zone of the DG for each condition (mean ± SEM, n = 3 to 6 sections per condition). The intensity plots were acquired as shown in c, the gray box highlights 10 μm of the subgranular zone, where the signal is found. (**c**) Example image illustrating the regions of interest (ROIs) used to acquire the intensity plots of GFP. The solid rectangle shows a ROI of 50 μm thickness across the granular zone. The dotted rectangle shows 10 μm of the subgranular zone of the DG, where NSPCs reside, and corresponds to the gray box in the graphs. (**d**) Overlay of the GFP intensity plots. The red line shows the SF protocol post-fixed with PFA at 25 °C, the blue line corresponds to GFP intensity in sections from a perfused brain. (**e**) The bar graph represents the width of the GFP intensity plot from the different conditions, (mean ± SEM, n = 3 to 6 sections per condition). Brain sections processed with the SF protocol and post-fixed with PFA at 25 °C show a wider curve compared to sections from perfused mice, suggesting a higher spreading of GFP fluorescence. *p < 0.001 (one-way ANOVA compared to perfused, n = 3 to 6 sections per condition). (**f**) Quantification of the area under the curve (AUC) in the 10 μm range where NPSCs reside (mean ± SEM, n = 3 to 6 sections per condition). SF-25 °C and DF-25 °C showed a significant decrease in AUC, *p < 0.05 (one-way ANOVA, followed by Holm-Sidak’s multiple comparisons, compared to perfused).

Surprisingly, with 4 °C PFA, the GFP signal was barely detectable, indicating that crosslinking of proteins was too slow to retain GFP, while nuclear staining was still present (Fig. 2b,c). When increasing the temperature of PFA to 30 °C or 37 °C, the signal of GFP appeared more confined to cell bodies (Fig. 2b,c) compared to 25 °C PFA (Fig. 2b,c) however still not as confined as in sections from a PFA-perfused mouse (Fig. 1a). Sections post-fixed with 30 °C PFA seemed to have the least spreading of GFP signal, leading to a similar percentage of GFP signal over the DG compared to sections from a PFA-perfused mouse (Fig. 2c), however, due to variability, this treatment was not significantly different from the 25 °C and 37 °C fixation. To visualize intensity differences among the different conditions, false color intensity mapping was applied to the GFP signal (Fig. 3a). Sections fixed with the DF protocol with 30 °C or 37 °C PFA showed more high intensity pixels compared to sections processed with the SF protocol or with the DF protocol using 25 °C PFA, indicating less GFP leakiness (yellow color, Fig. 3a). To quantify these differences, GFP signal intensity was measured, starting from the hilus side across the granular zone of the DG (50 μm, Fig. 3b–d). The intensity peaked in all the conditions in the sub-granular zone of the DG, where NSPCs reside (Fig. 3b–d). Given that NSPCs in the DG reside in a very defined location just below the granular zone, the GFP intensity curve should follow a Gaussian curve, with intensity fading the further away from the cell bodies. Leakiness of GFP beyond cell bodies would thus result in a wider intensity curve. Therefore, the width of the curve was used as an indicator of the leakiness (Fig. S1). Indeed, intensity plots from samples processed with the SF protocol were significantly wider compared to perfused condition (Fig. 3d,e), whereas the DF protocol did not change significantly the width of the curve, showing that DF without any drying step leads to a more confined signal compared to sections processed with SF. However, the improvement with PFA temperature, which was observable with false color intensity mapping (Fig. 3a), was not captured with this quantification (Fig. 3e).

To evaluate the improvement of the staining with the increasing temperature of PFA, we assessed the area under the curve (AUC) in the region where NSPCs reside (Fig. 3d, gray box). The AUC of samples processed with 25 °C PFA, either with SF or DF, showed a significant decrease compared to the perfused condition, while 30 °C and 37 °C were comparable to the perfused condition (Fig. 3f).

To test if this protocol was applicable to other tissues and other GFP-reporter models, brain and lungs from a Cx3Cr1-GFP reporter mouse were extracted and subjected to the same protocol as shown in Fig. 2a. Cx3Cr1 is expressed in microglia in the brain, and in tissue macrophages in peripheral organs, such as the lung (Fig. S2a). In sections of pre-fixed tissue, GFP co-localized perfectly with the ionized calcium binding adaptor molecule 1 (Iba1), a pan-macrophage marker. In snap-frozen sections however, the GFP signal was diffuse and not restricted to the shape of the microglia, as outlined with Iba1. The use of DF-PFA 30 °C improved the signal compared to SF-PFA, DF-PFA 25° and DF-PFA 37 °C (Fig. S2b). Similarly to the brain sections, pre-fixed lung sections showed a perfect co-localization of GFP with Iba1 (Fig. S2c), whereas GFP leakiness was also observed in snap-frozen lung tissue with various post-fixation protocols. Using the DF-PFA 30 °C protocol, the GFP signal was also improved in the lung tissue (Fig. S2c).

Summarizing these results, we here show that a DF protocol using warm PFA for post-fixation of snap-frozen, unfixed cryosections is improving the GFP staining compared to standard protocols.

## Discussion

Using reporter mice simplifies the visualization of proteins or cells of interest. Indeed, GFP has demonstrated to be helpful in such applications. Because GFP protein is easily lost from unfixed frozen sections, it is important to fix the specimen prior freezing samples^4^. However, fixation of the entire tissue before sectioning might not always be compatible with downstream applications.

Fixation procedures allow taking a snapshot of a dynamic tissue while maintaining tissue organization. Chemical fixatives are commonly used in research and diagnostics and act through different stabilization mechanisms. Methanol and acetone are fixatives that remove water from tissue, leading to a precipitation of proteins. Paraformaldehyde, as other aldehydes, cross-links proteins and other macromolecules^13^. Appropriate fixation is crucial for the correct detection of the protein of interest and optimal protocols depend on the downstream application. As chemical modifications can mask the epitopes or change the conformation of proteins, different fixation protocols can massively influence the detection of the protein of interest. Furthermore, permeabilisation after fixation is also influencing this detection and can vary according to the combination of fixation/permeabilisation used^14,15^. The relevance of this issue is nicely illustrated by the recent debate about the existence of adult neurogenesis in humans^16^. Depending on the fixation and tissue pre-treatment protocol for post-mortem samples, Moreno-Jiménez and colleagues could demonstrate that the immature neuron marker doublecortin (DCX) was either detectable or absent, which obviously leads to contrary conclusions^17^. Due to fixation artefacts, it has further been suggested that whenever possible, studies using fluorescent fusion proteins should also integrate live imaging to confirm results^18^.

In the case of detection of soluble GFP the problem starts even before fixation. Many researchers have accepted that GFP cannot be used in unfixed frozen tissue, because the protein leaks out of the cells due to damaged membranes during the thawing process. While Morris and colleagues could detect GFP in frozen sections prior any washing step, although the intensity was extremely low, the fluorescence was measured in the buffer after the washing steps, supporting the idea of GFP leakiness^6^. Such leakiness is less prominent with larger reporter proteins such as the tandem dimer Tomato (TdTomato), thus it was proposed to use TdTomato for applications requiring unfixed frozen tissue^6^. However, as many reporter organisms are engineered with GFP protein, switching to tdTomato is often not a feasible solution.

To overcome this problem, GFP has to be fixed before it can leak out, however a standard post-fixation of unfixed cryosections with paraformaldehyde is not sufficient to detect good quality GFP fluorescence, as we show here and others have previously reported^6,19^. Jockusch and colleagues managed to immobilize GFP protein in frozen skeletal muscles by exposing the cryosections to vapors of 37% formaldehyde at − 20 °C. Indeed, compared to control sections, GFP signal was detectable in cells expected to express it, even though the fluorescent signal appeared still diffuse^19^. While this study showed that GFP could be prevented from leaking out of unfixed tissue by specific post-fixation procedures, using formaldehyde vapors poses a significant health risk, as its inhalation is toxic and can lead to lung damage. Thus, a safer method is preferable.

Post-fixation of cryosections is usually performed after sections are placed on glass slides and allowed to air-dry, which was also the case for the studies mentioned above. By mounting frozen sections on glass, the difference in temperature allows the section to thaw and to stick on the glass. Our findings suggest that this step is also critical for GFP to diffuse out of the section. Whereas mounting the section on the glass slide is crucial to maintain tissue integrity of thin sections, the timing between the sectioning and the post-fixation can be modulated. We thus omitted the drying step to minimize GFP diffusion. This small modification already improved the signal, suggesting that GFP indeed leaks out during the warming up and drying steps. As the temperature of the fixative will contribute to the warming up of the section, we reasoned to use cold PFA to further minimize the temperature shift of the mounted section. To our surprise, this did not improve the signal, on the contrary, it worsened it. Similarly, mounting the section on cold glass had no beneficial effect either (data not shown). These data suggested that the rate of fixation was not quick enough when using cold PFA.

As the rate of penetration of PFA increases proportionally with its temperature^13^, we attempted to improve GFP signal by increasing the temperature of PFA. By this simple procedure, we were able to obtain a more confined and intense GFP signal in sections post-fixed with either 30 °C or 37 °C PFA compared to those treated with 25 °C PFA (Figs. 2b, 3), with a clear expression pattern in the subgranular zone of the DG. Whether increasing the temperature of other fixatives such as methanol of acetone would lead to an improvement of GFP fixation remains to be determined. By using another GFP-reporter model, the Cx3Cr1-GFP model, we could also observe GFP leakiness in microglia and macrophages when tissues were snap-frozen. Also in this case, we observed that DF-PFA 30 °C led to an improvement of the signal (Fig. S2). However, none of the tested protocols gave an equally defined signal as obtained with tissue from transcardially-perfused mice. Interestingly, another fixative called glyoxal has recently been proposed to be a relevant alternative to PFA, preserving many cellular structures when used as a direct fixative^20^. Whether or not glyoxal would work as a post-fixative to preserve soluble GFP in snap frozen tissue remains to be examined.

Taken together, our novel protocol allows using unfixed snap-frozen tissue as a starting material, but still being able to detect GFP. Thus, different techniques can be performed in adjacent sections. As these sections are only a few microns apart, data obtained from the different modalities can be overlaid afterwards. This is for instance interesting for combining metabolomics data obtained through IMS with immunohistological analyses using GFP reporter mice.

## Methods

### Animals

Six-week old Nestin-GFP^11^ and Cx3Cr1-GFP^12^ reporter mice were used for the experiment. The Veterinary office of the canton Vaud, Switzerland, approved all animal studies and all experiments were performed in accordance with relevant guidelines and regulations.

### Tissue processing

To obtain fixed brain tissue of Nestin GFP reporter mice, transcardial perfusion with 0.9% saline solution followed by 4% cold PFA was performed. The brain was taken out and post-fixed overnight at 4 °C in 4% PFA, and subsequently stored in 30% sucrose/phosphate buffer saline (PBS) solution. 20 μm thick section were cut on a cryo- microtome (Leica, Switzerland).

For snap frozen preparations, mice were killed by cervical dislocation. The brain was taken out, and directly immersed in dry ice-cold isopentane (Sigma-Aldrich, 270,342) and afterwards kept at -80 °C until cryosectioning. Frozen tissues were mounted onto a specimen holder with a drop of Tissue-Tec OCT (Fisher Scientific, 12678646) and cut with a Cryo-microtome (Leica, CM3050S) at 12 µm thickness. Sections were placed on SuperFrost Plus slides (Fisher Scientific, 10149870). Slide were allowed to air dry for 5 min, or not, depending on the protocol (see Fig. 1c,e for details).

All sections were post-fixed by immersion in fixatives for 15 min, either at room temperature (25 °C, RT) or at the indicated temperatures. As mentioned in Figs. 1c,e and 2a, different fixatives were used: 100% cold methanol (kept at − 20 °C prior fixation), cold acetone (kept at − 20 °C prior fixation) and 4% PFA which had different temperature (4 °C, 25 °C, 30 °C or 37 °C). Post-fixation with methanol or acetone was done in the cryostat chamber (− 20 °C). To maintain PFA at 4 °C, sections were put in a container with cold PFA, and incubated on ice. Sections processed with PFA at 25 °C were done at room temperature. PFA was warmed up to 30 °C or 37 °C in a water bath and the fixation was also performed at the corresponding temperature. All sections were washed 2 times 10 min with PBS.

For Cx3Cr1-GFP tissues, a heterozygous Cx3cr1GFP/ + mouse was killed by cervical dislocation after a lethal injection of pentobarbital. Brain and lungs were harvested and cut into two pieces. One was directly immersed in dry ice-cold isopentane (Sigma-Aldrich, 270342), the other one was post-fixed overnight at 4 °C in 4% PFA and subsequently stored in 30% sucrose/phosphate buffer saline (PBS) solution. Similarly to Nestin-GFP brain, tissues were cryo-cut at 12 μm thickness and processed as described here above and in Figs. 1c,e and 2a.

### Immunohistochemistry

For both perfused or fresh frozen and post-fixed samples, sections were directly blocked for 1 h with blocking buffer (0.2% Triton-X (Sigma-Aldrich, X100), 3% Donkey serum (Merck, S30) in Tris-buffered saline (TBS, 50 mM Tris–Cl, pH 7.4, 150 mM NaCl), and incubated with the indicated primary antibodies in blocking buffer at 4 °C overnight. The following primary antibodies and dilutions were used: Chicken-anti GFP (1:500, abcam, ab13970), goat anti-SOX2 (1:500, R&D, AF2018) and rabbit anti-Iba1 (1:1,000, Fujifilm Wako, 019–19,741).

Sections were washed 3 times in TBS for 10 min and incubated with secondary antibodies at a concentration of 1:250 (Alexa Fluor 488 AffiniPure Donkey Anti-Chicken, JacksonImmuno Research, 703–545-155, and Alexa Fluor 594 AffiniPure Donkey Anti-Goat, JacksonImmuno Research 705-585-147) in blocking buffer at least 1 h at room temperature, protected from light. Slides were washed 2 times 10 min with TBS. Nuclei were stained with DAPI (1:5,000, Sigma-Aldrich, D9542) for 5 min. After other 3 washes of 10 min each, slides were mounted using self-made polyvinyl alcohol (PVA, Sigma P8136) mounting medium with 1, 4-diazabicyclo[2,2,2]octane (DABCO, Sigma D27802).

### Imaging and analysis

Pictures were acquired with a confocal microscope (LSM-710, Zeiss). For the 20 µm thick fixed sections, images of 12 µm depths were acquired with a Z-step size of 1 μm with a 20 × objective. For the 12 µm thick cryosections, images were acquired with a Z-step size of 1 μm with a 20 × objective and a maximum projection was used for the following analyses.

To determine the percentage of GFP signal on the sections, analyses were carried out using Fiji software (ImageJ). Based on DAPI channel, DGs were contoured, and GFP images were thresholded, and the percentage of the area covered by GFP within the DG contour was acquired.

To determine the profile of GFP signal in the DG, ten to thirteen regions of interest (ROIs) of 138.4 μm (200 pixels) width and 50 μm (72.2 pixels) height, per image, were drawn based on DAPI channel. The ROIs were placed so that they were parallel to the granular zone of the DG and covered the width of it (Fig. 3c). For each ROI, a plot profile for GFP signal intensity was acquired using Fiji (ImageJ) software. This tool displays a two-dimensional graph where the x-axis represents the distance along the ROI, in this case 50 μm height and the y-axis is the averaged pixel intensity through the selection. For each picture, the average of ROIs was calculated in order to have only one plot profile per picture. The values were further normalized to the intensity measured at 50 μm (set as the background intensity) and expressed as percentage. The GFP signal is expected to be highly concentrated in specific cells and sharply diminish outside this region. Thus, the intensity plots follow a Gaussian curve, with narrower curves indicating more localized signal. Therefore, the following equation was used to assess the GFP intensity curve.

$$y=b+a \cdot {e}^{\left(-\frac{1}{2}\cdot {\left(\frac{x-m}{s}\right)}^{2}\right)}+\varepsilon $$

The parameters obtained with this model are the width (s parameter), the center of the curve (m parameter), the baseline of the intensity (b), the amplitude of the curve (a) and ε stands for the observed errors between the measured points and the fitted curve. The width represents the spreading of the fluorescence into the tissue. The smaller it is, the more confined GFP fluorescence is at a specific point.

Non-linear regression was done with RStudio (R version 3.5.1). Examples of fitted curved are displayed in Fig. S1.

The area under the curve (AUC) of the GFP intensity within 10 μm (gray box, Fig. 3d) was calculated using Graphpad Software.

### Statistics

Graphs and statistical analyses were performed using the software GraphPad Prism 8 software (Graphpad Software Inc. version 8.0.2).

One-way ANOVA was performed followed by Holm-Sidak’s multiple comparisons tests.

Results were expressed as means ± standard error of the mean (SEM). Statistical significance is indicated with asterisks for p-values < 0.05.

## Supplementary information

Supplementary information.
