# Supplementary material for: A novel protocol to detect green fluorescent protein in unfixed, snap-frozen tissue
Source: Sci Rep. 2020 Sep 4;10:14642. doi: 10.1038/s41598-020-71493-x (PMC7474079; doi:10.1038/s41598-020-71493-x)
Supplement: Supplementary file 1 — Supplementary information. [file 41598_2020_71493_MOESM1_ESM.pdf]

A novel protocol to detect green fluorescent protein in unfixed, snap-frozen tissue.

Valentina Scandella, Rosa Chiara Paolicelli, Marlen Knobloch.

All the authors are affiliated to the Department of Biomedical Sciences, University of Lausanne, 1005 Lausanne, Switzerland

Supplementary information

2 Figures and Figure legends

Supplementary figure 1

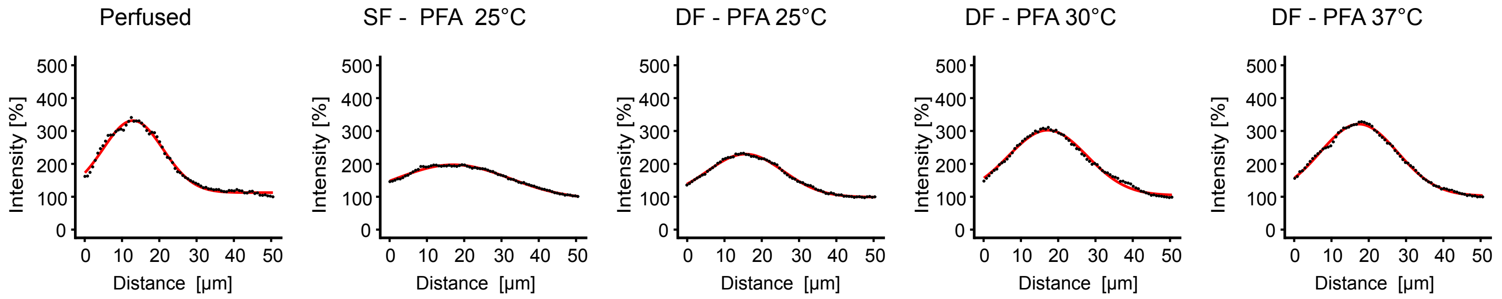

Supplementary Figure 1: Examples of fitted curves for the different conditions to assess the width of the curves shown in Fig. 3b. Black dots represent the measured values and the red line shows the fitting curve.

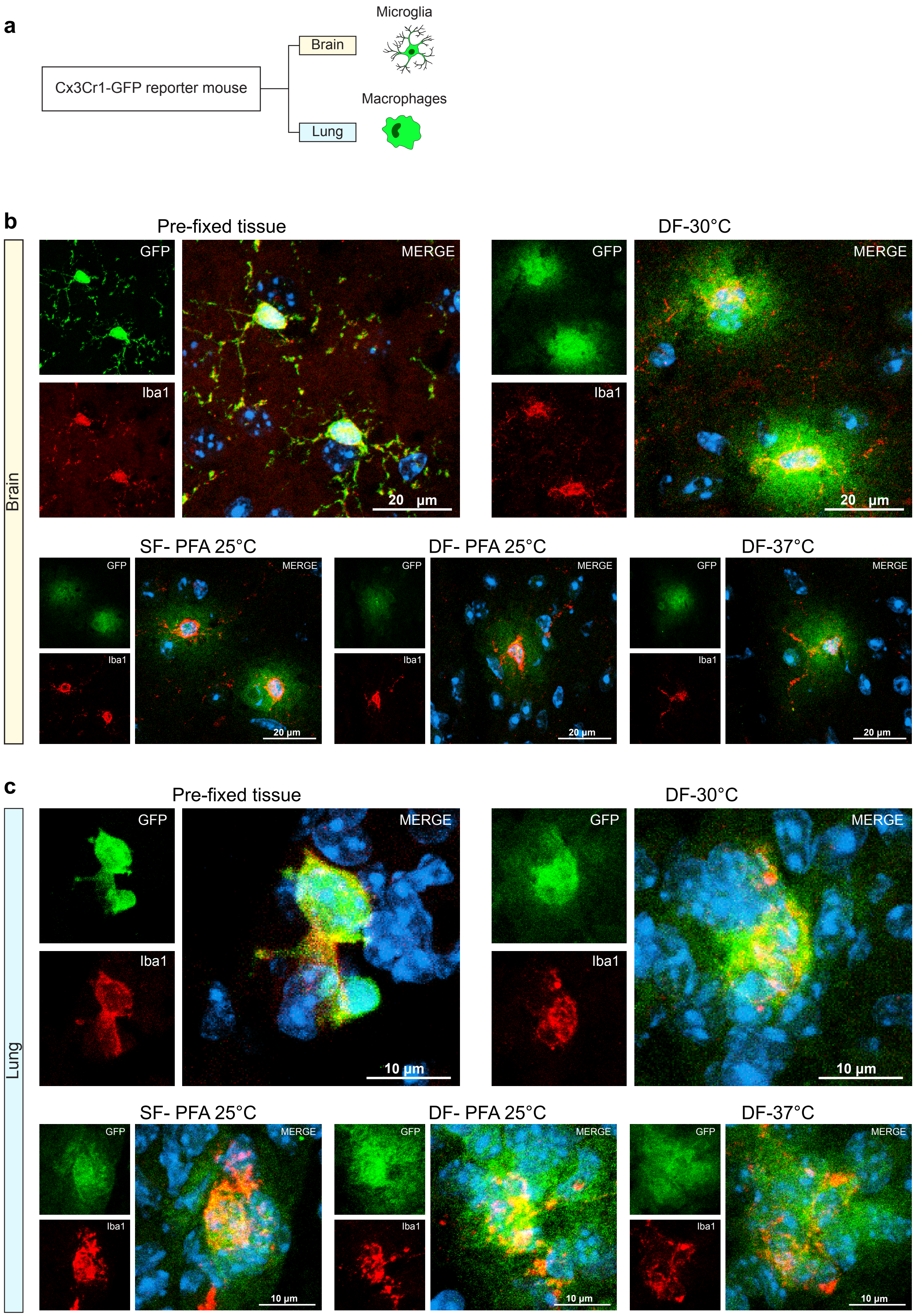
a
Cx3Cr1-GFP reporter mouse
Brain
Lung
Microglia
Macrophages
b
Brain
Pre-fixed tissue
DF-30°C
GFP
Iba1
MERGE
20 µm
SF- PFA 25°C
DF- PFA 25°C
DF-37°C
c
Lung
10 µm

Supplementary Figure 2: Comparison of SF and various DF protocols described in Fig. 1 and Fig.2 on snap-frozen brain and lung sections of a Cx3Cr1-GFP reporter mouse. **(a)** Cx3Cr1-GFP labels microglia in the brain and tissue macrophages, as found for instance in the lung. **(b)** Co-staining of GFP (green) and the pan-macrophage marker Iba1 (red) on brain sections from a Cx3cr1-GFP mouse show that Cx3cr1-GFP^+ microglia co-localize with $Iba1^+$ microglia. Snap frozen brain sections were processed with the different PFA fixation protocols shown in Fig.1 and Fig.2. The DF protocol with 30°C PFA post-fixation gave the best results, however did not reach the quality of pre-fixed tissue **(c)** Co-staining of GFP and Iba1 in pre-fixed or snap frozen lung sections from a Cx3cr1-GFP mouse. Snap frozen lung section were processed with the different PFA fixation protocol as in (b). For all images, a maximum projection of a confocal stack with the individual channels and a merge is shown. DAPI is used to mark nuclei.
